# Supplementary figures and images for: Fish species identification on low resolution—a study with enhanced super-resolution generative adversarial network (ESRGAN), YOLO and VGG-16
Source: PeerJ Comput Sci. 2025 Apr 30;11:e2860. doi: 10.7717/peerj-cs.2860 (PMC12192931; doi:10.7717/peerj-cs.2860)

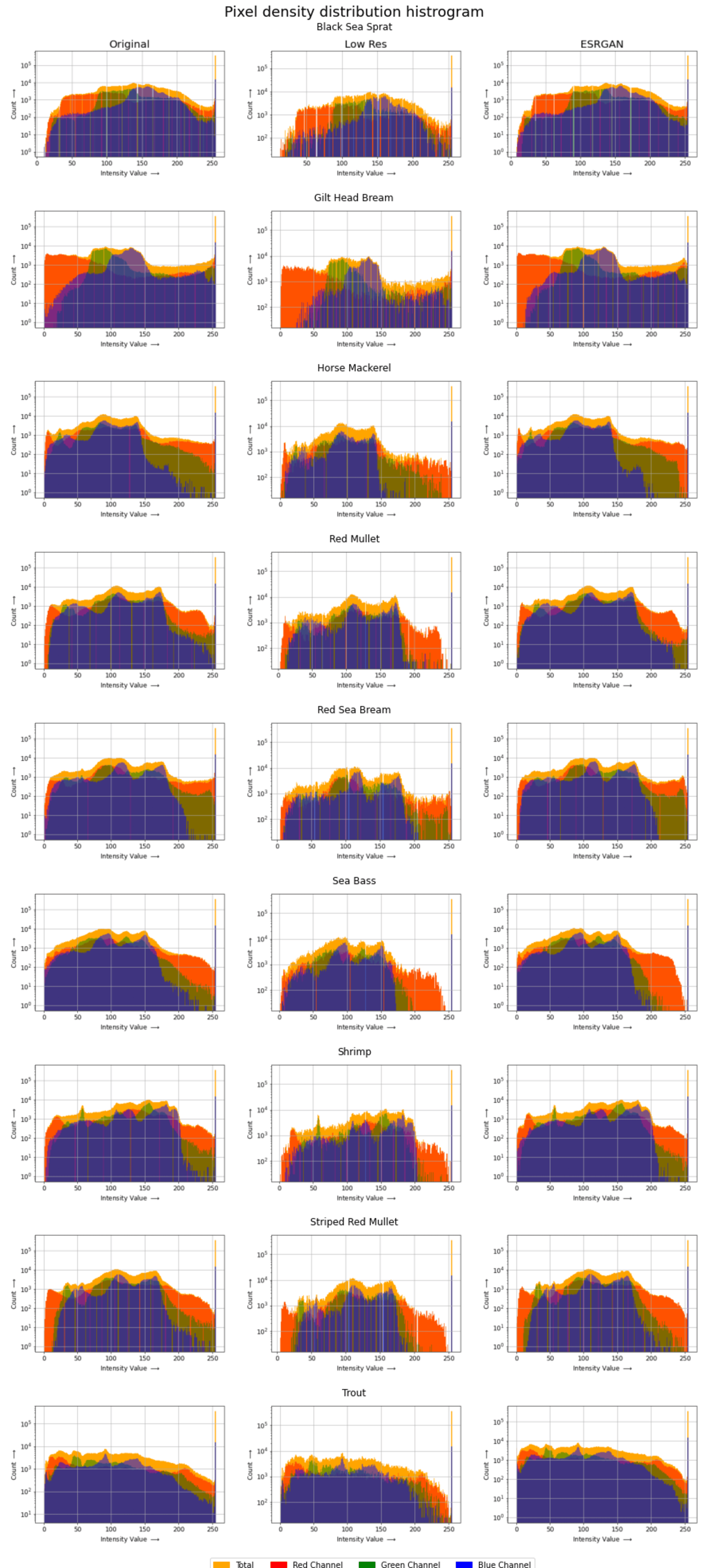

Supplement: Supplemental Information 1 — A pixel density distribution histogram for red, green, blue, and all colors combined (represented by yellow color) for pixel intensity ranging from 0-255. For all the species, it can be observed that the peaks of all the graph colors for low-resolution images are uneven. Still, for the original and ESRGAN-generated images, the peaks are smoother. Also, the ESRGAN-generated images and Original images have a higher number of pixels for each intensity level for each color compared to the low-resolution image, which is evident from the fact that the low-resolution image had been reduced by 1/4th of the original image. However, original and ESRGAN-generated super-resolved images have no generic differences, but some variations can still be observed, which change according to the target picture. For example, in graphs of Horse Mackerel, during the intensity range of 225 to 250, few blue bars are visible in the original image, which is missing in ESRGAN-generated images. Similar patterns can also be observed in many other fish species as well. [file peerj-cs-11-2860-s001.png]
